# Supplementary material for: Bubble-water/catalyst triphase interface microenvironment accelerates photocatalytic OER via optimizing semi-hydrophobic OH radical
Source: Nat Commun. 2024 Mar 15;15:2346. doi: 10.1038/s41467-024-46749-z (PMC10943107; doi:10.1038/s41467-024-46749-z)
Supplement: Supplementary file 1 — Supplementary Information [file 41467_2024_46749_MOESM1_ESM.pdf]

Supplementary Information

Bubble-water/catalyst triphase interface microenvironment accelerates photocatalytic OER via optimizing semi-hydrophobic OH radical

Guanhua Ren[1†], Min Zhou[1†], Peijun Hu[1,2], Jian-Fu Chen[1] & Haifeng Wang[1*]

[1]State Key Laboratory of Green Chemical Engineering and Industrial Catalysis, Centre for Computational Chemistry and Research Institute of Industrial Catalysis, East China University of Science and Technology, Shanghai 200237, China

[2]School of Chemistry and Chemical Engineering, Queen's University Belfast; Belfast, UK

[†]These authors contributed equally: Guanhua Ren, Min Zhou

*Corresponding author: hfwang@ecust.edu.cn

Supplementary Figures

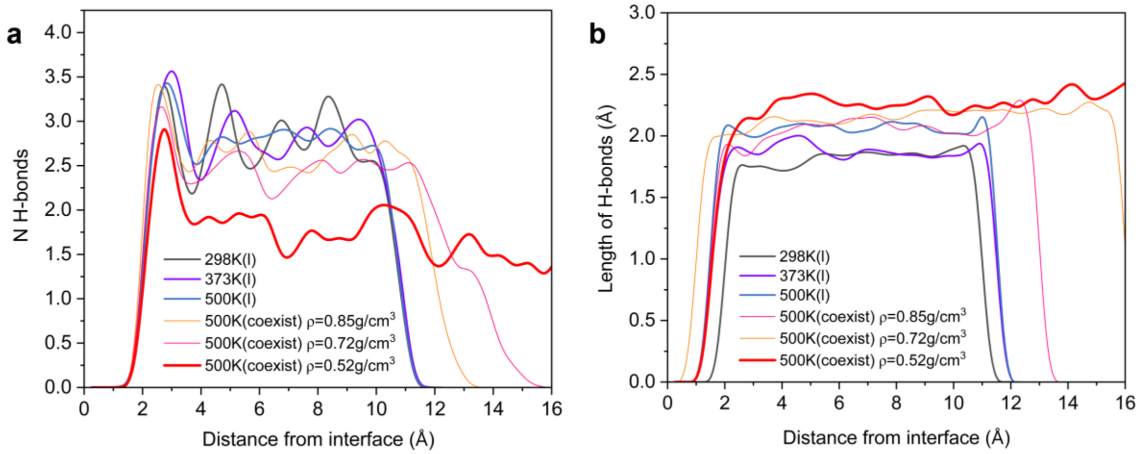

Supplementary Fig. 1 | Comparison of the distributions of H-bonds per water molecule along the interface. a Distribution of the average number of H-bonds per water molecule. b Distribution of the average length of H-bonds per water molecule.

Note: In terms of the structural configuration, water molecules in the typical liquid phase interact with adjacent molecules to form six-, five- or four-member ring structures. As the temperature increases, the interface environment undergoes a transition into a more disordered hydrogen bonding network. In the case of 500 K (coexist), the density of water molecules is already lower than that of the typical liquid water. Particularly under the condition depicted in Fig. 1f of the main text, the volume occupied by water molecules has almost doubled, resulting in a significant decrease in water density of 48%. Supplementary Fig. 1 reveals the numbers and lengths of hydrogen bonds as a function of the distance from the interface in six cases from the statistical analysis. As the volume increases, the average number of H-bonds per water molecule gradually decreases. Particularly, at the first and second water layers of the liquid/catalyst interface, there is a significant reduction in the number of H-bonds at 500 K (coexist). This demonstrates that the hydrogen bond network is destroyed to some extent once the liquid-gas transition begins to occur. Furthermore, as the H-bonds are disrupted, the average length of the hydrogen bonds also increases (Supplementary Fig. 1b).

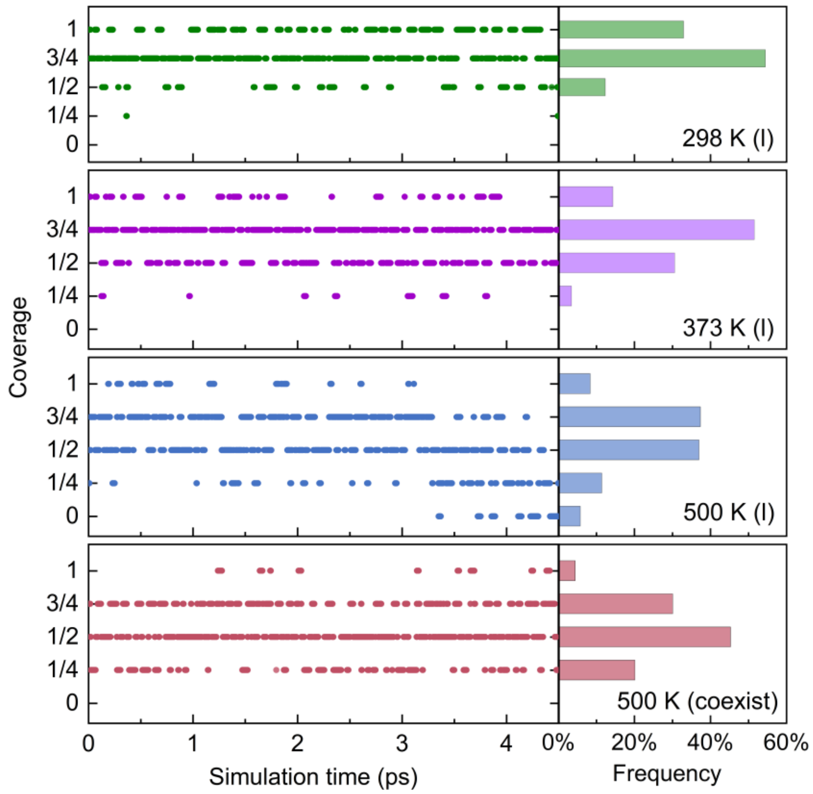

Supplementary Fig. 2 | Statistical analysis of the MD simulations under different conditions. The left shows the coverage of surface H_2O molecules adsorbed at Ti_{5c} site evolving with simulation time, while the right is the histogram of the resulting probability distribution.

Notes: The statistical analysis of simulation with coverage of surface water molecules at different conditions was obtained from the last ~4 ps duration of each AIMD. Fig. 1a shows a structure of the liquid water/rutile TiO_2(110) interface under standard conditions with an experimental water density of 1g/ml. It can be seen that water molecules close to the TiO_2(110) surface bind to the Ti_{5c} sites with the oxygen atoms of the water molecules in the first layer at ~2 Å from the surface. We determine the Ti-O_{water} bond length 2.32 Å as the chemisorbed bond length (equal to the length at the gas condition) and count the coverage changing with simulation time. As Supplementary Fig. 2 shows, at 298K (l), although the water molecules adsorb/desorb dynamically on/from the surface in the AIMD simulations, the coverage of surface water molecules is always approximately in the range of 0.75 ML (four molecules per surface TiO_2(110) equal to 1 ML/surface coverage). When the temperature is increased but still below the phase transition temperature, the interfacial water network distribution is slightly affected by the temperature. At 373 K (l), the first layer water molecules are more likely to desorb from the surface, resulting in the water coverage being in the range of 0.5 ML to 0.75 ML. As for the 500 K (l), the coverage is

dominant between 0.5 ML and 0.75 ML, but the probability of 0.25 ML has increased. For comparison, the vaporization process of liquid water under a high-pressure condition at 500 K was simulated (Fig. 1f). As the height of the total water layer increases, some water molecules in the first layer are more likely to desorb from the surface, resulting in the water coverage being in the range of 0.25 ML to 0.75 ML, where the 0.5 ML becomes the main coverage.

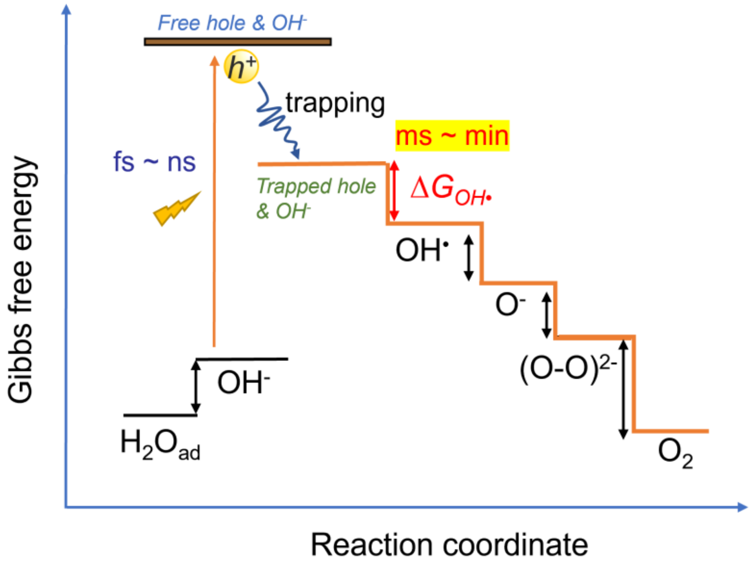

Supplementary Fig. 3 | The reaction mechanism of OER and the related free energy diagram.

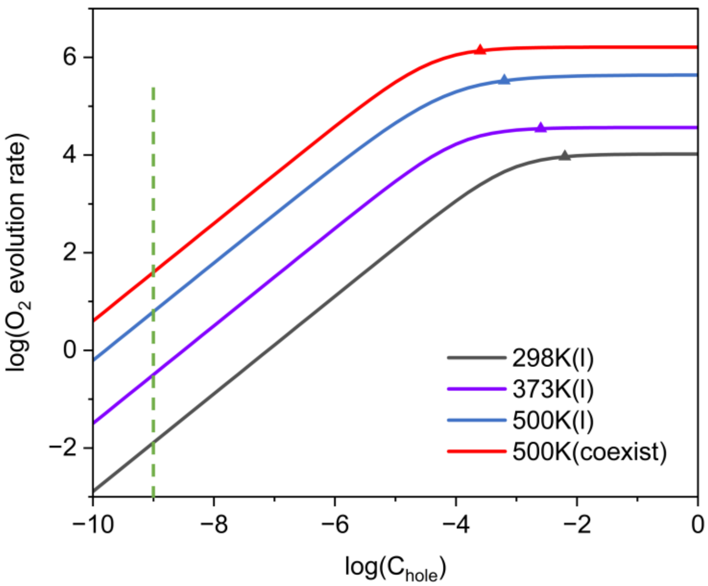

Supplementary Fig. 4 | The logarithmic plots of the OER rates at different water/TiO_2(110) interfaces as a function of hole concentration.

Note: The hole-electron recombination and the intensity of light irradiation are important for the overall photoactivity since they directly affect the hole concentration on the surface. In Supplementary Fig. 4, we highlight that the concentration of holes reaching the surface impacts significantly on the OER rate. Specifically, we investigated how the hole concentrations affect the OER rate under four typical conditions (298 K(l), 373 K(l), 500 K(l) and 500 K(coexist)) with the hole concentration ranging from 10^{-10} to 1 ML, as illustrated in Supplementary Fig. 4. It can see the following: (*i*) the OER rates increase significantly from the very low concentration of surface-reaching hole and reach a plateau at a certain threshold value of the hole concentration. The threshold values become gradually smaller from 298 K(l)→373 K(l)→500 K(l)→ 500 K(coexist), indicating the relatively small dependence of hole-concentration on the liquid-vapor coexisting interface environments. (*ii*) Comparison with the other three conditions, it consistently exhibits better OER activity under the 500 K (coexist) condition with varying hole concentration, aligning with the corresponding activity trend described in Fig. 1h of the main text.

Here we take the case of 500 K(coexist) as an example to further illustrate the effect of hole concentration. When the hole concentration is in the range from 10^{-10} to 10^{-4} ML, an increase in hole concentration leads to a higher rate of OER. When the hole concentration exceeds 10^{-4} ML, the OER rate reaches a plateau and the rate-determining step shifts, because at a higher hole concentration, the formation of $^{\bullet}$OHt via trapping holes at the terminal OHt− becomes easier and is no longer the rate-determining step. Moreover, the presence of micro-bubbles under the liquid-vapor coexisting conditions slows down the

water dissociation reaction and make it earlier to be the rate-determining step (relative to the 298 K(l), 373 K(l) and 500 K(l) conditions). In this case, increasing the concentration of surface-reaching holes cannot further enhance the reaction rate.

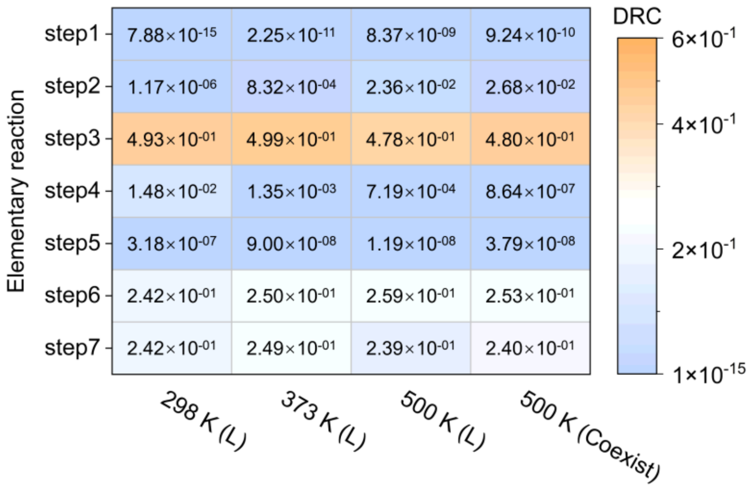

Supplementary Fig. 5 | Degree of rate control (DRC) for each elementary reaction under different conditions. The color gradient of the orange-blue scale marks the relative magnitude of the DRC value.

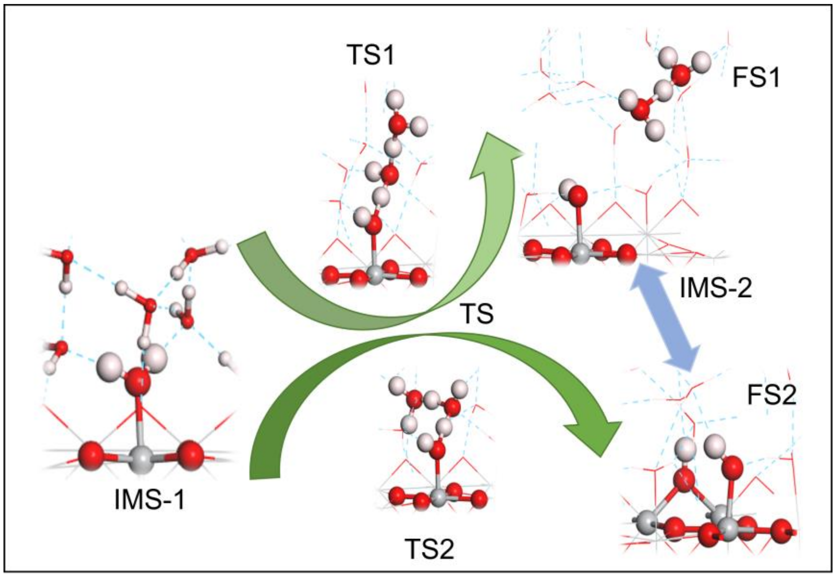

Supplementary Fig. 6 | Schematics for two possible pathways of H_2O_{ad} deprotonation into a terminal hydroxyl OH_t^- and a H^+ in solution (step 2: $H_2O_{ad} \rightarrow OH_t^- + H^+(sol)$).

Note: At the transition state (TS), the detaching H^+ bounds to the nearby water in solution. Next, the H-O bond of H_2O_{ad} breaks and forms the Zundel-like ($H_5O_2^+$) structure with nearby water in the near interface. Then, the ($H_5O_2^+$) structure through a series of catch-and-abandon-like proton transfer chains forms a stable ($H_5O_2^+$) structure in the liquid (FS1) or transfer the proton to the bridge oxygen site (O_{br}) on the surface (FS2). The proton in the O_{br} site would further detach and migrate to the liquid by a similar way of proton transfer.

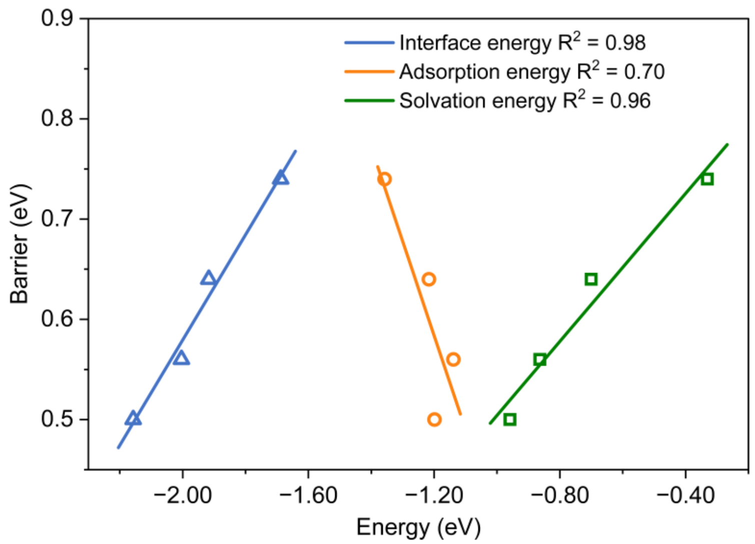

Supplementary Fig. 7 | The relationships between the deprotonation barrier of H_2O_{ad} and the interface energy, adsorption energy, and the solvation energy of H_2O_{ad} at the interfaces of 298 K (l), 373 K (l), 500 K (l), 500 K (coexist), respectively.

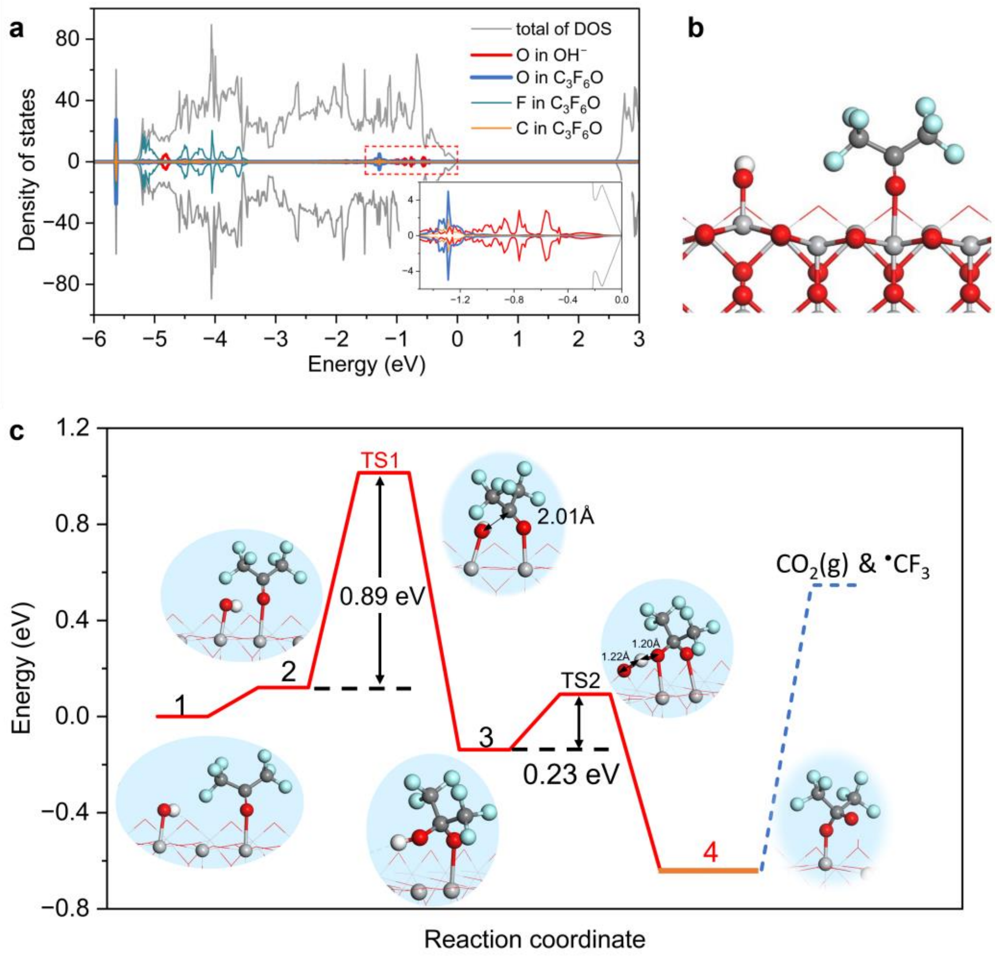

Supplementary Fig. 8 | Illustration of the stability of hexafluoroacetone during the photocatalytic process. Density of states (DOS) of $TiO_2(110)$ with OH_t^- and hexafluoroacetone adsorbed on the terminal Ti_{5c} (a) and the corresponding geometry structure (b). **c** Energy profile of possibility of the hexafluoroacetone conversion assisted by the surface $OH^\bullet$ radicals in the reaction condition.

Note: It is worth mentioning that the stability of the hydrophobic substance in the *in situ* photocatalytic condition is an important issue for the realistic application. Here we use the hexafluoroacetone as a proof of concept to demonstrate the effect of hydrophobic microenvironment on modulating the photocatalytic OER, and some basic reasons using hexafluoroacetone are as follows: (*i*) it contains -CF_3 groups, which has strong hydrophobic property; (*ii*) it has a C=O group, which allows it to adsorb onto the catalyst surface; moreover, its adsorption energy is relative lower than that of water molecule, and it will not excessively occupy the reaction sites. (*iii*) it is thermodynamically stable and does not chemically react with water molecules; for example, the enthalpy change of the reaction: $H_2O + (CF_3)_2C{=}O \rightarrow (CF_3)_2CHO^\bullet + {}^\bullet OH$, is strongly endothermic (>> 2 eV); (*iv*) regarding

the photoexcitation effect, we evaluated the ability of C_3F_6O itself to trap holes, and we found that C_3F_6O has a significantly weak hole trapping capability (HTC) of about -0.24 eV, which is much weaker than that of OH^- (-0.91 eV). As shown in the density of states (DOS) in Supplementary Fig. 8a, the highest occupied state of O in C_3F_6O is much lower, as low as -1.30 eV, compared to the O of OH^- (located at -0.5 eV). Therefore, C_3F_6O itself is less likely to be photoexcited under photocatalytic condition, making it difficult to decompose.

Further, we examined the possibility of oxidative conversion of hexafluoroacetone assisted by the surface $OH^\bullet$ radicals to check the stability of hexafluoroacetone under the photocatalytic reaction condition, as illustrated in Supplementary Fig. 8c. Specifically, as $(CF_3)_2C{=}O$ itself cannot not be self-activated via trapping hole (as shown in Supplementary Fig. 8a), the coupling of $(CF_3)_2C{=}O$ with $OH^\bullet$ radical ($(CF_3)_2C{=}O^* + {}^*OH^\bullet \rightarrow (CF_3)_2C(OH)O^*$, i.e., process 1→3) as the prerequisite reaction to activate $(CF_3)_2C{=}O$ was calculated, including the possible dehydrogenation conversion of $(CF_3)_2C(OH)O^*$ to the carboxylate species ${}^*(CF_3)_2COO^-$. As shown in Supplementary Fig. 8c, the whole process (1→4) is exothermic but gives an effective barrier as high as 1.01 eV; this indicates that hexafluoroacetone could be oxidized by $OH^\bullet$ radical in thermodynamics, but kinetically unfavorable at typical room temperature. In other words, hexafluoroacetone should be relatively stable, although the long-term stability could be an issue. We emphasize that it is the importance to consider the long-term stability when selecting the other hydrophobic substance for realistic application in further studies.

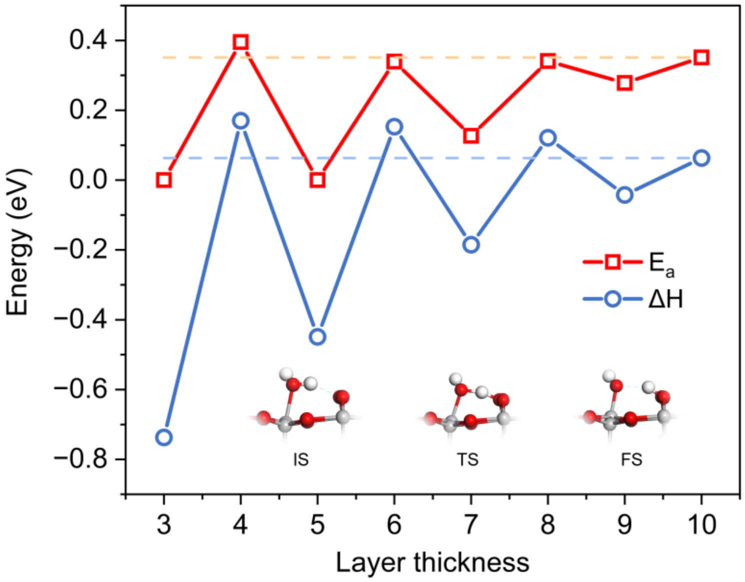

Supplementary Fig. 9 | Comparison of energy barrier (E_a) and enthalpy change (ΔH) for water dissociation on rutile TiO_2(110) versus the number of TiO_2 layers. The horizontal dotted line gives the value of 10-layer slab. Inserted structures are the initial state (IS), transition state (TS), and final state (FS), respectively. Grey, red, and white balls represent Ti, O, and H atoms, respectively.

Note: The TiO_2(110) surface exhibits layer-dependent structural and energetic oscillations, predominantly characterized by odd-even oscillation with respect to the number of layers in a slab. This observation is largely determined by the atomic structure of TiO_2(110) and is a long-standing concern in the literature. To mitigate the impact of this issue, and also to ensure that the project progresses within a reasonable timescale, there are three reasons for us to choose the 4-layer p(4×1) model:

Firstly, the use of 4-layer p(4×1) model yields reasonably accurate results within a manageable computational time frame. As illustrated in Supplementary Fig. 9, we compared the results for the water dissociation barrier (E_a) and enthalpy change (ΔH) on the rutile TiO_2(110) surface against the number of TiO_2 layers. We can see that the 4-layer model gives similar results to those from the 10-layer slab. Also, the results from the 4-layer model are close to those from the 6-layer and 8-layer models. It is important to note that although the use of thicker slabs unquestionably gives more reliable results, the computational workload increases exponentially with thickness. Therefore, within a reasonable margin of error, the 4-layer model is the more economically viable choice.

Secondly, the 4-layer *p*(4×1) model has been consistently used in the published theoretical and computational work, as supported by a number of studies in the field of photocatalysis[1-4]. Specifically, we compared the energy profile of water dissociation on the

4-layer $p(4\times1)$ surface with the experimental data reported by Wang et al.[5], demonstrating the agreement between the calculated results (E_a=0.37 eV and ΔH=0.16 eV) and experimental data (E_a=0.36 eV and ΔH=0.035 eV). This alignment underscores the reliability of our chosen model in capturing the energetics of this process.

Thirdly, one of the focuses in this current work is on accurately simulating the aqueous state at different conditions and obtaining the reliable reaction energies. According to the reference[6], the water density distributions are very similar for the different slabs from 4 to 16 layers and overlap each other almost completely. This result further confirms that the 4-layer structure can be used to simulate a realistic solid-liquid interface system.

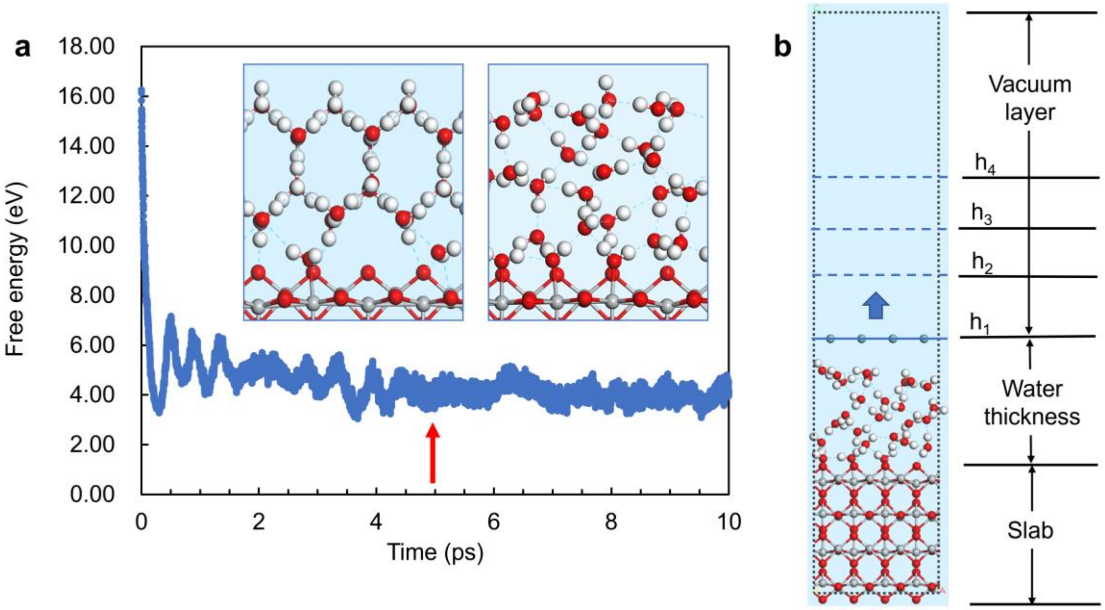

Supplementary Fig. 10 | Models of the water/TiO_2(110) system operating in the AIMD simulation. a Energy profile of AIMD simulation for water/TiO_2(110) system, in which the geometries inserted are the front and side views of the system containing initial water structure (ice-like). The red arrow indicates roughly the time that the energy starts to oscillate around a constant. **b** Schematic of water/TiO_2(110) system under different conditions. Grey: Ti; red: O; white: H; green: Ar.

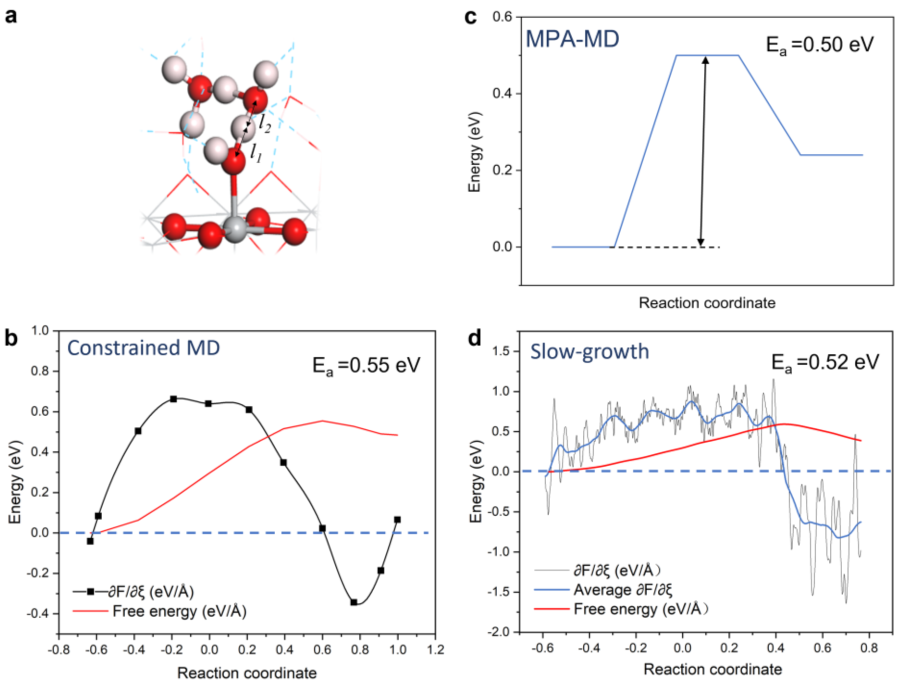

Supplementary Fig. 11 | Energy comparison of H_2O deprotonation process obtained by MAP-MD, the constrained MD, and the slow-growth methods. a The definition of the reaction coordinate, equal to the difference between H_{ad}-O_{ad} bond distance l_1 in H_2O_{ad} and the H_{ad}-O_n distance l_2 (O_n is the oxygen atom of nearest water in liquid). **b-d** Energy profiles of H_2O deprotonation process at $H_2O/TiO_2(110)$ interface using the constrained MD method (b), the MPA-MD method (c), and the slow-growth method (d).

Notes: Firstly, the constrained MD method was established on the basis of thermodynamic integration of the free-energy gradient within the statistical mechanics framework[7-10]. It is a well-accepted approach to accurately calculate the free energy change (including reaction barriers). In the constrained MD method, the Nosé-Hoover thermostat and NVT ensemble were usually used. The free energy difference (ΔF) between state (1) and (2) is obtained based on the identification of the gradient $(\partial F/\partial \xi)_{\xi*}$ determined at each constrained distance $\xi(1\rightarrow2)$, where the $(\partial F/\partial \xi)_{\xi*}$ stands for the statistical average of $(\partial F/\partial \xi)$, and ξ is the reaction coordinate typically corresponds to a key geometric parameter linking state (1) and (2). As shown in Supplementary Equation (1), ΔF can be computed by integrating free energy gradients over ξ along the reaction path (1⟶2):

$$\Delta F_{1\rightarrow2} = \int_{\xi(1)}^{\xi(2)} \left(\frac{\partial F}{\partial \xi}\right)_{\xi*} d\xi \qquad (1)$$

In our constrained MD simulation for calculating the barrier of H_2O deprotonation process, the reaction coordinate ξ is equal to the difference between HO_{ad}-H_{ad} bond distance (l_1) in H_2O_{ad} and the H_{ad}-O_n distance l_2 (O_n is the oxygen atom of the nearest

interface water molecule), that is $\xi = l_1 - l_2$ as illustrated in Supplementary Fig. 11a. As shown in Supplementary Fig. 11b, the ξ value is stretched gradually from -0.65 to 1.0 Å during the H_2O deprotonation process. For each fixed ξ, we performed long-time MD simulation in NVT ensemble (T =298 K) until quasi-equilibrium state was achieved. All the interatomic forces along the reaction coordinate, which corresponds to the free energy gradients, can be obtained statistically. Then the free energy change can be obtained by integrating these free energy gradients.

Overall, the constrained MD is an accurate but very time-consuming method to calculate the complex OER network at the water/TiO_2(110) interface. Alternatively, we developed the MPA-MD method to deal with the aqueous systems, and the reaction energetics were thoroughly tested in our previous work, including the reaction barriers, to verify the reliability of the MPA-MD method[2]. As shown in Supplementary Table 7, five kinds of aqueous interface reactions were compared between MPA-MD and the state-of-the-art constrained MD method. The MPA-MD method gives very similar results to the constrained MD method in all cases. In particular, for the H_2O deprotonation reaction, both methods give the comparable barriers (0.50 versus 0.55 eV; see Supplementary Fig. 11c), demonstrating the feasibility of MPA-MD method in studying the OER mechanism at the water/TiO_2(110) interface.

Secondly, the slow-growth based AIMD method is another effective way to calculate the free energy profile, which has been successfully used to capture the varying H-bonding networks during the aqueous reaction[11-14]. Compared to the common constrained MD method, the reaction coordinate (ξ) in the slow-growth method is changed linearly from state (1) to state (2) with a constant and very small transformation velocity $\dot{\xi}$. This is the biggest difference from the common constrained MD approach. The resulting free energy difference needed to perform a transformation from state (1) to state (2) can be computed as:

$$\Delta F_{1\to 2} = \int_{\xi(1)}^{\xi(2)} \left(\frac{\partial F}{\partial \xi}\right)_{\xi^*} d\xi = \lim_{\dot{\xi}\to 0} \int_{\xi(1)}^{\xi(2)} \left(\frac{\partial F}{\partial \xi}\right) \cdot \dot{\xi}\, dt \qquad (2)$$

where F is the free energy calculated at the coordinate ξ which evolves with t, and $\partial F/\partial \xi$ is calculated along the MD trajectory by the SHAKE algorithm[15]. Overall, the slow-growth method has a relatively lower computational cost at a similar (or slightly worse) accuracy than the common constrained MD method, which was therefore also used to quickly determine the reaction coordinate prior to the common constrained MD method.

Specifically, for the H_2O deprotonation process, $\xi = l_1 - l_2$ was similarly chosen as the collective variable (CV) as shown in Supplementary Fig. 11d, and a very small value $\partial \xi$ of 0.0005 Å was used in practice for each MD step; It is worth mentioning that the shorter step size for the describing slow-growth process along the reaction coordinate was also

tested, which verified the validity of the $\partial\xi$ value. Notably, the slow growth approach is available in the VASP code, and we used the standard exponentially weighted moving average (EWMA) method to process the average value of $\partial F/\partial\xi$. An example of the raw output data from VASP and the integrated free energy profile is demonstrated in Supplementary Fig. 11d. The barrier of H_2O deprotonation process with this approach is 0.52 eV, which is close to the value obtained from MPA-MD method (0.50 eV).

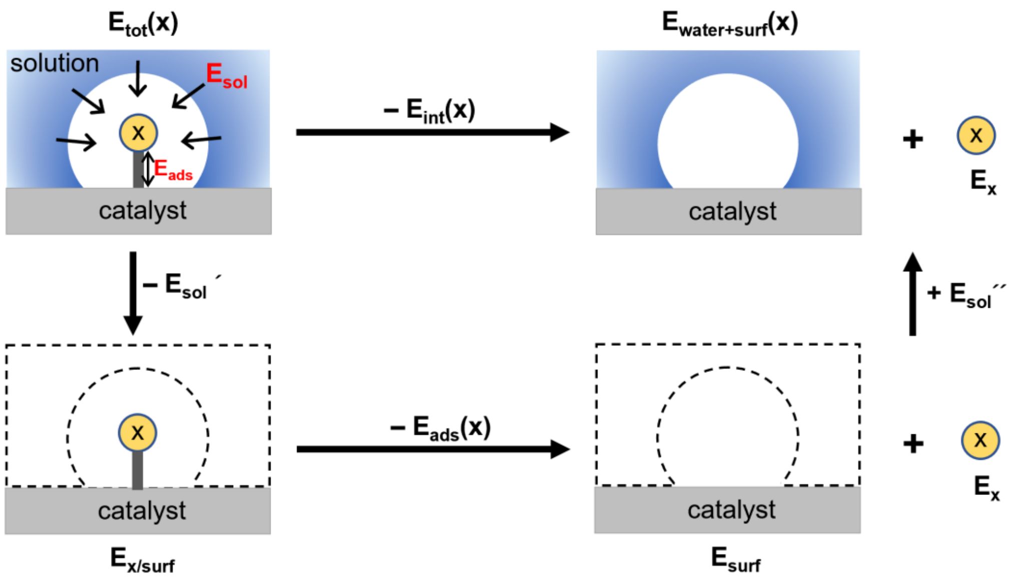

Supplementary Fig. 12 | Schematics of the interfacial energy calculation procedures. A Haber-cycle was designed to calculate the solvation energy contriction (E_{sol}(x)) of the adsorbate x itself, which is $E_{sol}(x) = E_{sol}{}' - E_{sol}{}'' = E_{int}(X) - E_{ads}(X)$.

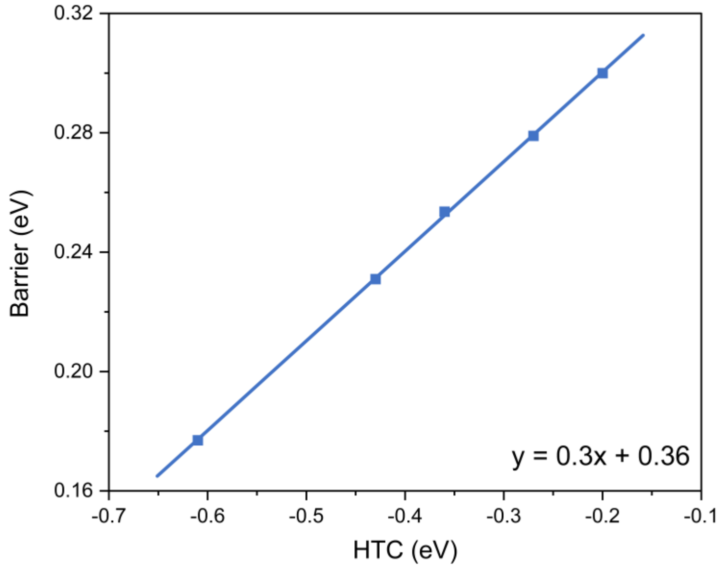

Supplementary Fig. 13 | Brønsted–Evans–Polanyi (BEP) relationship for the hole transfer to the surface OH_t^- species at different conditions.

Supplementary Tables

Supplementary Table 1 | The optimized average Ti-O bond lengths $l_{(Ti\text{-}O)}$ of adsorbed H_2O at different conditions. Unit: Å.

Condition	$l_{(Ti\text{-}O)}$
298 K (l)	2.18
373 K (l)	2.22
500 K (l)	2.24
500 K (coexist)	2.39
Gas	2.32

Supplementary Table 2 | Reaction barriers (E_a) and free energies (ΔG) of elementary steps *i* in photocatalytic OER process for solving kinetic modeling. Unit: eV.

Steps	Reaction equations	298 K (l)		373 K (l)		500 K (l)		500 K (coexist)		Hydrophobic interface	
		ΔG	E_a	ΔG	E_a	ΔG	E_a	ΔG	E_a	ΔG	E_a
1	$H_2O(sol) + {}^* \rightarrow {}^*H_2O$	-0.16	/	0.15	/	0.39	/	0.18	/	-0.20	/
2	${}^*H_2O \rightarrow {}^*OH_t^- + H^+(sol)$	0.24	0.50	-0.25	0.56	-0.48	0.64	-0.54	0.74	-0.14	0.62
3	${}^*OH_t^- + h^+ \rightarrow {}^{*\bullet}OH_t$	-0.20	0.30	-0.27	0.28	-0.36	0.25	-0.61	0.18	-0.43	0.23
4	${}^{*\bullet}OH_t \rightarrow {}^*O_t^- + H^+(sol)$	-0.53	0.41	-0.44	0.43	-0.44	0.36	-0.34	0.48	-0.35	0.35
5	${}^*O_t^- + {}^*O_t^- \rightarrow {}^*O_2^{2-} + {}^*$	-0.21	0.24	-0.14	0.27	0.03	0.32	-0.07	0.27	-0.02	0.22
6	${}^*O_2^{2-} + h^+ \rightarrow {}^*O_2^-$	-1.82	0.30	-1.77	0.28	-1.69	0.25	-1.54	0.18	-1.50	0.23
7	${}^*O_2^- + h^+ \rightarrow O_2(aq) + {}^*$	-2.45	0.30	-2.29	0.28	-2.46	0.25	-1.81	0.18	-2.11	0.23

Notes: The OER process starts from the water adsorbing on the Ti-row sites of TiO_2(110) (step 1), and the H_2O_{ad} deprotonates in solution to form a Zundel-like ($H_5O_2^+$) structure and produce the OH_t^- at the Ti-row site (step 2). Next, the generated OH_t^- could be further oxidized into ${}^\bullet OH_t$ via a hole trapping (step 3). Furthermore, the ${}^\bullet OH_t$ deprotonates, yielding the O_t^- radical on the Ti-row site in a similar proton transfer mode with the H_2O_{ad} (step 4). The newly produced O_t^- radical could couple with another adjacent O_t^- radical on the Ti-row site, generating O_2^{2-} (step 5). In this case, the O_2 evolutes via the trapping of two successive holes to oxidize O_2^{2-} (step 6 and 7).

It is interesting to find that the interfacial environment also remarkably influences the enthalpy change of the water dissociation process. Specifically, the enthalpy change of water dissociation at 298 K (l) is ~0.24 eV, suggesting that the dissociated water is more likely to reverse to H_2O molecule rather than further oxidized. While at the states of 373 K (l) and 500 K (l), the enthalpy changes are reduced to ~-0.25 eV and ~-0.48 eV, respectively. It is noteworthy that the enthalpy change of deprotonation under the 500 K (coexist) condition is -0.54 eV, far surpassing the values of other interfaces, implying that the temperature can regulate the deprotonation processes by changing the interface environments. The enthalpy change of this step at 500 K (coexist) remarkably facilitates the forward reaction. This suggests that the appropriate coexisting environment of liquid and vapor can increase the driving force of the forwarding reaction by decreasing the final state energy (more exothermic).

Supplementary Table 3 | Valence charges (Bader charge) for the surface $^{\bullet}OH_t$ and OH_t^- intermediates at the different interfaces. Unite: |e|.

	$^{\bullet}OH_t$			OH_t^-		
	O	H	Ti	O	H	Ti
300K (l)	-1.059	1.000	2.457	-1.555	1.000	2.459
400K (l)	-1.058	1.000	2.463	-1.600	1.000	2.470
500K (l)	-1.054	1.000	2.446	-1.595	1.000	2.488
500 K (coexist)	-1.033	1.000	2.458	-1.630	1.000	2.461
Hydrophobic interface	-1.056	1.000	2.466	-1.548	1.000	2.466
Gas	-1.009	1.000	2.463	-1.489	1.000	2.446

Supplementary Table 4 | The adsorption energy E_{ads}(x), solvation energy E_{sol}(x), and interface energy E_{int}(x) of H_2O and $^{\bullet}OH_t$ intermediates at different conditions.

		298 K (l)	373 K (l)	500 K (l)	500 K (coexist)	gas
H_2O	E_{ads}	-1.20	-1.14	-1.22	-1.36	-1.44
	E_{sol}	-0.96	-0.86	-0.70	-0.33	0.00
	E_{int}	-2.16	-2.00	-1.92	-1.69	-1.44
$^{\bullet}OH_t$	E_{ads}	-0.87	-0.87	-0.91	-1.10	-1.13
	E_{sol}	-0.95	-0.87	-0.71	-0.49	0.00
	E_{int}	-1.81	-1.74	-1.62	-1.59	-1.13

Supplementary Table 5 | Parameters of the water/TiO_2(110) models using in the AIMD simulation at different temperatures (T= 298, 373, and 500 K). The density of water in different systems can be estimated by the formula: ρ_{H2O}=(N×M_{H2O})/ (N_A×a×b×h), where N is the number of H_2O molecules, corresponding to value of 26 in these systems; a and b is the length and width of the slab (a:11.84 Å, b: 6.50 Å), and h is the actual thicknesses of the water structure.

Picture	T (K)	N	h (Å)	ρ (g/cm^3)	States	Label
Fig. 1a	298	26	10.03	1.00	*l*	298 K (l)
Fig. 1b	373	26	10.03	1.00	*l*	373 K (l)
Fig. 1c	500	26	10.03	1.00	*l*	500 K (l)
Fig. 1d	500	26	11.92	0.85	coexist	500 K (coexist)
Fig. 1e	500	26	14.14	0.72	coexist	500 K (coexist)
Fig. 1f	500	26	19.36	0.52	coexist	500 K (coexist)
Fig. 4b	298	18	10.03	1.00	*l*	Hydrophobic interface

Supplementary Table 6 | Entropy change (TΔS) and zero-point-energy changes (ΔZPE) of H_2O adsorption (step 1) and O_2 desorption steps (step 7) at different reaction temperatures. Unit: eV.

Steps	298 K (l)		373 K (l)		500 K (l)		500 K (coexist)		Hydrophobic interface	
	TΔS	ΔZPE	TΔS	ΔZPE	TΔS	ΔZPE	TΔS	ΔZPE	TΔS	ΔZPE
1	-0.22	0.05	-0.34	0.02	-0.45	-0.01	-0.45	0.02	-0.22	0.00
7	0.63	-0.04	0.83	-0.04	1.14	-0.03	1.14	-0.02	0.63	-0.04

Supplementary Table 7 | Comparison of reaction barriers using MPA-MD and the constrained MD method.

Reaction in solutions	E_a (eV) (MPA-MD)	E_a (eV) (constrained MD)
$Pt(NH_3)_2Cl_2 + H_2O \rightarrow Pt(NH_3)_2Cl(H_2O)^+ + Cl^-$	0.78	0.75 (exp. 0.84-1.07)[16]
$Na_nCl_n \rightarrow Na_nCl_{n-1}^+ + Cl^-$	0.15	0.11[17]
$O_2 \rightarrow 2O^*$ on Pt (111)	0.43	0.39
$O_2 + H^* \rightarrow OOH^*$ on Pt (111)	0.60	0.58
$H_2O^* \rightarrow OH^- + H^+$ on TiO_2 rutile(110)	0.50	0.55

References

1. Wang, Z. et al. Localized excitation of Ti(3+) ions in the photoabsorption and photocatalytic activity of reduced rutile TiO_2. *J. Am. Chem. Soc.* **137**, 9146-9152 (2015).
2. Wang, D., Sheng, T., Chen, J., Wang, H.-F. & Hu, P. Identifying the key obstacle in photocatalytic oxygen evolution on rutile TiO_2. *Nat. Catal.* **1**, 291-299 (2018).
3. Zhang, J., Peng, C., Wang, H. & Hu, P. Identifying the role of photogenerated holes in photocatalytic methanol dissociation on rutile TiO_2(110). *ACS Catal.* **7**, 2374-2380 (2017).
4. Zhou, M. & Wang, H. Optimally selecting photo- and electrocatalysis to facilitate CH_4 activation on TiO_2(110) surface: localized photoexcitation versus global electric-field polarization. *JACS Au* **2**, 188-196 (2022).
5. Wang, Z.-T. et al. Probing equilibrium of molecular and deprotonated water on TiO_2 (110). *PNAS* **114**, 1801-1805 (2017).
6. Wen, B., Calegari Andrade, M. F., Liu, L. M., & Selloni, A. Water dissociation at the water–rutile TiO_2(110) interface from ab initio-based deep neural network simulations. *PNAS*, **120**, e2212250120 (2023).
7. Sprik, M. & Ciccotti, G. Free energy from constrained molecular dynamics. *J. Chem. Phys.* **109**, 7737-7744 (1998).
8. Carloni, P., Sprik, M. & Andreoni, W. Key steps of the cis-platin-DNA interaction: density functional theory-based molecular dynamics simulations. *J. Phys. Chem. B* **104**, 823-835 (2000).
9. Bucko, T. Ab initio calculations of free-energy reaction barriers. *J. Phys. Condens. Matter* **20**, 064211 (2008).
10. Peng, C., Chen, J., Hu, P. & Wang, H. Molecular adsorption kinetics: nonlinear entropy–enthalpy loss quantified by constrained AIMD and insights into the adsorption-site determination on metal oxides. *J. Phys. Chem. C* **125**, 10974-10982 (2021).
11. Woo, T. K., Margl, P. M., Blöchl, P. E., & Ziegler, T. A combined Car-Parrinello QM/MM implementation for ab initio molecular dynamics simulations of extended systems: application to transition metal catalysis. *J. Phys. Chem. B.* **101**, 7877-7880 (1997).
12. Jarzynski, C. Nonequilibrium equality for free energy differences. *Phys. Rev. Lett.* **78**, 2690-2693 (1997).
13. Oberhofer, H., Dellago, C., & Geissler, P. L. Biased sampling of nonequilibrium trajectories: can fast switching simulations outperform conventional free energy calculation methods? *J. Phys. Chem. B* **109**, 6902-6915 (2005).
14. Zhao, X. & Liu, Y. Unveiling the active structure of single nickel atom catalysis: critical roles of charge capacity and hydrogen bonding. *J. Am. Chem. Soc.* **142**, 5773-5777 (2020).
15. Ryckaert, J.-P., Ciccotti, G. & Berendsen, H. Numerical integration of the cartesian equations of motion of a system with constraints: molecular dynamics of n-alkanes. *J. Comput. Phys.* **23**, 327-341 (1997).
16. Song, T. & Hu, P. Insight into the solvent effect: a density functional theory study

of cisplatin hydrolysis. *J. Chem. Phys.* **125**, 091101 (2006).

17. Liu, L. M., Laio, A. & Michaelides, A. Initial stages of salt crystal dissolution determined with ab initio molecular dynamics. *Phys. Chem. Chem. Phys.* **13**, 13162–13166 (2011).
